# On Cancer, COVID-19, and CT Scans: A Monocentric Retrospective Study

**DOI:** 10.3390/jcm9123935

**Published:** 2020-12-04

**Authors:** Francesca Martini, Andrea D’Alessio, Federico Bracchi, Daniela Di Mauro, Anna Fargnoli, Marco Motta, Cristina Giussani, Marco Meazza Prina, Giovanni Gobbin, Monica Taverna

**Affiliations:** Department of Internal Medicine and Onco-Haematology, Policlinico S. Marco, 24040 Bergamo, Italy; dr.dalessio@gmail.com (A.D.); fede.bracchi@hotmail.it (F.B.); daniela_dimauro@virgilio.it (D.D.M.); annafargnoli@gmail.com (A.F.); marco.motta89@gmail.com (M.M.); crigiusi84@gmail.com (C.G.); marco.meazzaprina@gmail.com (M.M.P.); giovanni.gobbin@gmail.com (G.G.); monica.taverna@fastwebnet.it (M.T.)

**Keywords:** COVID-19 infection, CT scan, oncological patients

## Abstract

Background The use of computed tomography (CT) for coronavirus disease 2019 (COVID-19) diagnosis in an area of northern Italy with a high incidence of severe acute respiratory syndrome coronavirus 2 (SARS-CoV-2) infection may have identified more patients with this disease than RT-PCR in the very early onset of the COVID-19 pandemic. Methods We retrospectively reviewed 148 chest CT scans of oncological patients who were referred to the Radiological Unit of Policlinico S. Marco from 1 February 2020 to 30 April 2020, during the COVID-19 outbreak in Bergamo area. In parallel, we analyzed RT-PCR tests of these 148 patients. Results Among 32 patients with a diagnosis of COVID-19, 17 patients were asymptomatic or had mild symptoms (53.1%), while 15 developed severe disease (46.8%). The incidence of SARS-CoV-2 infection was 22.9%, the mortality rate was 18.8%. We did not find any correlation between disease severity and age, sex, smoking, or cardiovascular comorbidities. Remarkably, patients who were on treatment for cancer developed a milder disease than patients who were not on treatment. Conclusions The acceptance of CT-defined diagnoses in COVID-19 high-incidence areas like Bergamo region highlighted a larger oncological population affected by COVID-19 than RT-PCR, in particular, asymptomatic and mildly symptomatic patients, because only symptomatic patients underwent nasopharyngeal swabbing at the onset of the COVID-19 pandemic. We observed that patients actively treated for their cancer had a milder disease, in agreement with previous studies that suggested a protective role of immunosuppression. Admittedly, the sample of patients in our study was heterogeneous regarding the oncological disease, their prognosis, and the type of treatment; therefore, other studies are needed to confirm our data.

## 1. Introduction

According to three recently published Chinese studies, patients with cancer have a higher risk of COVID-19 [1,2,3]. Liang’s study was criticized because it concluded that patients with cancer had a higher risk of COVID-19 on the basis of a higher percentage of patients with cancer in the COVID-19 cohort than in the overall population. However, the incidence of COVID-19 in patients with cancer would be a more valid index to determine whether patients with cancer have an increased risk of COVID-19 [4]. Moreover, it is difficult to determine if the worse outcome of COVID-19 infection in patients with cancer is related to other confounding factors such as history of smoking, older age, comorbidities rather than to their cancer history [5,6].

Finally, the reports of a benign course of COVID-19 in immunocompromised patients suggests that immunosuppression can be a “double-edged sword”: adaptive immune response can contribute to either recovery or disease [7,8].

Data on patients with COVID-19 who have cancer have been recorded by a number of large registry-based studies in order to identify potential prognostic factors for mortality and severe illness.

The report from CCC19 (The COVID-19 and Cancer Consortium) identified increased age, male sex, smoking status, number of comorbidities, Eastern Cooperative Oncology Group performance status of 2 or higher, active cancer, and receipt of azithromycin plus hydroxychloroquine as factors associated with decreased 30-day all-cause mortality. Race and ethnicity, obesity status, cancer type, type of anticancer therapy, and recent surgery were not associated with mortality [9].

According to UKCCMP (UK Coronavirus Cancer Monitoring Project) report, mortality from COVID-19 in cancer patients appears to be principally driven by age, gender, and comorbidities. There is no evidence that cancer patients on cytotoxic chemotherapy or other anticancer treatments are at an increased risk of mortality from COVID-19 disease compared with those not on active treatment [10].

A registry has been analyzed to understand the impact of COVID-19 specifically on thoracic cancer patients (TERAVOLT): in multivariable analysis, only smoking history was associated with increased risk of death in this type of cancer. The data suggest high mortality and low admission to intensive care in patients with thoracic cancer [11].

Moreover, the data recorded from Memorial Sloan Kettering Cancer Center revealed that age ≥65 years and treatment with immune checkpoint inhibitors (ICI) within 90 days were predictors for hospitalization and severe disease in cancer patients affected by COVID-19, while receipt of chemotherapy within 30 days and major surgery were not. Overall, COVID-19 illness is associated with higher rates of hospitalization and severe outcomes in patients with cancer [12].

According to the report from the Gustave Roussy Cancer Centre, age of over 70 years, smoking status, metastatic disease, cytotoxic chemotherapy, and an Eastern Cooperative Oncology Group score ≥2 at the last visit were the strongest determinants of increased risk of death. However, in multivariable analysis, the Eastern Cooperative Oncology Group score remained the only predictor of death [13] Overall, the impact of cancer type and treatment on COVID19 outcomes and the best oncological treatment strategy have not been consistently elucidated yet.

The diagnosis of COVID-19 can be challenging: some sources consider chest CT findings more sensitive than RT-PCR (nasopharyngeal swabbing) in detecting COVID-19 [14,15,16,17], despite less specificity, as imaging findings for CODIV-19 overlap with those of other types of viral pneumonia [18].

From February 2020 through April 2020, the COVID-19 pandemic has ravaged across the Lombardy Region of Northern Italy, in particular in Bergamo town. This is the economy driver of Italy and one of the most productive regions in Europe, with strong international links and a high-density population. As of 30 March 2020, among the 101,739 cases diagnosed, 42,161 were registered in Lombardy (Dipartimento di Protezione Civile press release 30 March 2020).

Considering the context of emergency disease, the acknowledgment of COVID-19 diagnosis based on chest CT imaging abnormalities, clinical and laboratory findings in a region with a high prevalence of the infection (as northern Italy has been at the very early onset of the pandemic) may have identified more patients with COVID 19 infection, in particular asymptomatic and mildly symptomatic patients, because only symptomatic patients underwent nasopharyngeal swabbing at the onset of the COVID-19 pandemic [19]. In fact, CT findings in patients with COVID 19 have been deeply described even in asymptomatic patients [20]. Admittedly, normal CT scans do not exclude SARS-CoV-2 infection, but according to the rapid evolution of COVID-19 pneumonia, it is important to follow up CT findings at different timepoints if COVID-19 is clinically suspected. In Italy, chest CT has commonly been used to monitor the progression and complications of the infection, rather than as a potential adjunct for the diagnosis of COVID-19.

## 2. Methods

We retrospectively reviewed chest CT scans of actively treated oncological patients and of cancer patients in follow-up after treatment or in a watch-and-wait follow-up, who were referred to the Radiological Unit of Policlinico S. Marco from 1 February 2020 to 30 April 2020, during the COVID-19 outbreak in Bergamo area.

In total, 148 CT scans were acquired with and/or without contrast medium injection. Reconstructed images were displayed on an ICIS view workstation and interpreted by a team of specialists experienced in thoracic radiology. Comparisons with prior patients’ scans were made when available. Decisions were reached by consensus.

Chest CT typically showed bilateral ground-glass opacities (GGOs) in 28 patients (87.5%), bilateral patchy shadowing in 1 patient (3%), and bilateral interstitial abnormalities in 1 patient (3%). Eight patients presented bilateral pulmonary consolidations in combination with one of the previously described CT features (25%). Infiltrates that could be associated with cancer metastases or radiation pneumonitis were ruled out. We retrospectively reviewed RT-PCR tests (sensibility and specificity 95%) of the patients who underwent CT scan in the same period of time.

The incidence of COVID-19 was calculated as the ratio between the number of cancer patients with a diagnosis of COVID-19 (CT scan suspicious of viral infection and/or positive RT-PCR test) and the total number of cancer patients who underwent a CT scan in the period from 1 February to 30 April.

The mortality rate of COVID-19 was calculated as the ratio between the number of patients whose death was related to viral pulmonary infection and the total number of cancer patients with a diagnosis of COVID-19 (CT scan suspicious of viral infection and/or positive RT-PCR test) from 1 February to 30 April.

Chi-square and Fisher’s exact tests were used to compare categorical variables; *p* < 0.05 was considered to be statistically significant. Yates’s correction was applied when needed.

Continuous variables were shown as median, and percentages were presented for categorical variables.

Severity score of disease was defined according to SIIARTI stage: the disease is defined as mild if the stage is I-II, severe if the stage is ≥III.

## 3. Results

**Figure 1:**
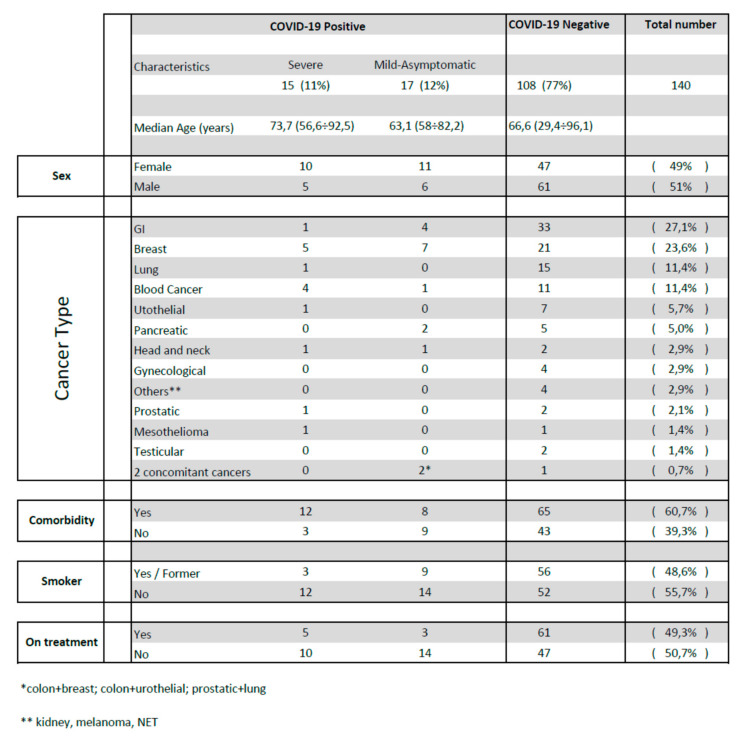
Clinical characteristics of cancer patients with severe, mild-asymptomatic COVID-19 infection and without infection.

Among the 148 scans of 140 patients (8 patients underwent more than one CT scan in the observed period), we identified 32 cases whose imaging findings were suggestive of COVID-19 (median follow time 27 days), (Table 1 and Figure 1).

The incidence of COVID-19 in our group of patients was 22.9%.

The mortality rate was 18.8% (6 patients died, among 32 with COVID-19).

Among 32 patients with COVID-19, 11 patients were asymptomatic (34.3%), 6 had mild symptoms (18.7%), while 15 developed severe disease (46.8%);14 patients needed hospital admission, and 3 of them needed intensive care unit (ICU) admission (9.3%), according to disease severity.

Eleven patients were asymptomatic. Nevertheless, they had CT scan features that were strongly suggestive of COVID-19. Four of them underwent nasopharyngeal swabbing before a clinical visit at the hospital after a median of 7–10 days from the CT scan: only one was SARS-CoV-2-positive. RT-PCR tests were not performed at the same time of CT scans, because COVID-19 was a casual finding in asymptomatic patients. One of the patients was not tested by RT-PCR, but her household members resulted to be infected (diagnosis made by RT-PCR).

Six patients had mild symptoms. One of them did not refer to the clinician for nasopharyngeal swabbing. The other five patients underwent RT-PCR tests at the onset of symptoms: four of them were SARS-CoV-2-positive, while one was negative. Two patients with RT-PCR positivity and mild symptoms had negative CT scans. However, in these two cases, CT scan was performed after symptoms remission for cancer follow-up.

Fifteen patients developed severe disease: all of them underwent CT scan and RT-PCR test concomitantly at the onset of the symptoms; 12 patients had positive CT scan and RT-PCR, while 3 had negative RT-PCR; of the latter, two were admitted to the hospital and proved negative for atypical pneumonia.

The median age of the patients with severe COVID-19 was higher (73.9 years) than those of patients with mild-asymptomatic COVID-19 (63.1 years) and of non-infected patients (66.6 years), but there was no statistically significant difference between patients older than 65 years and patients younger than 65 years in terms of incidence (*p* = 0.9), disease severity (*p* = 0.07 and mortality (*p* = 0.3).

In our group, more women than men were infected (*p* = 0.04), but there was no statistically significant difference between men and women considering disease severity (*p* = 1) and mortality (*p* = 0.6).

There was no significant difference of COVID-19 incidence, mortality, and disease severity between smoker (current and former) and non-smoker cancer patients (*p* = 0.1; *p* = 0.07; *p* = 0.1 respectively).

There was no significant difference of COVID-19 incidence, mortality, and disease severity between patients with cardiovascular (CV) comorbidities and patients without CV comorbidities (*p* = 0.6; *p* = 0.1 and *p* = 0.1 respectively).

There was no significant difference of COVID-19 incidence between cancer patients actively treated and patients who were not on cancer treatment at the time of their CT scan (*p* = 0.6). However, actively treated patients had a milder clinical picture and a lower mortality rate than patients who were not on treatment (odds ratio (OR) = 0.06 *p* = 0.002 and OR = 0.07 *p* = 0.018, respectively).

This study has a limitation because all 6 deceased patients had positive RT-PCR, while 10 of the 26 alive patients had no swabbing performed, so it is not possible to know for sure if they were affected. However, from February 2020 through April 2020, the COVID-19 pandemic has ravaged across the Lombardy Region of Northern Italy, in particular in Bergamo town. As of 30 March, 2020, among the 101,739 cases diagnosed in Italy, 42,161 were registered in Lombardy (Dipartimento di Protezione Civile press release 30 March). Therefore, considering the high prevalence of the infection in Bergamo area and the fact that all patients in the study had a relevant exposure history (household members affected by COVID 19-related pneumonia confirmed by RT-PCR or highly suspected on the basis of clinical, laboratory, and radiological features), we could assume that COVID-19 diagnosis can be based on chest CT imaging abnormalities and clinical features, even in the absence of an RT-PCR test.

## 4. Discussion

The incidence of COVID-19 in our group of patients was 22.9%. The mortality rate was 18.8% (6 patients died among 32 patients with COVID-19).

It is difficult to make comparisons of incidence and mortality rate between cancer patients and non-oncological patients, considering that a CT scan cannot be used as a screening tool for the general population. Consequently, we are not able to know at the moment how many asymptomatic or mildly symptomatic non-oncological people are infected. A recent published single-center retrospective study reported an infection rate of 2.7% among 1380 cancer patients, and those with the severe/critical disease corresponded to 54.1%. However, in this study, COVID-19 diagnosis was made by RT-PCR or an antibody test for SARS-CoV-2 [6].

We assume that in an area with a high incidence of SARS-CoV-2 infection and in the context of emergency, COVID-19 can be diagnosed by CT scan even if it is not confirmed by RT-PCR, when clinical and epidemiological features are compatible. Moreover, negative results of tests for atypical pneumonia made in parallel can support the diagnosis of COVID-19.

CT scan as a screening tool cannot be used for the general asymptomatic population, due to the health effects of the employed ionizing radiations. Combining imaging features with clinical and laboratory findings could facilitate the early diagnosis of COVID-19 pneumonia, in particular when the RT-PCR test is negative. The repetition of PCR testing should be driven by characteristic pneumonia features on CT as well as by clinical features in patients who initially had negative nucleic acid test results. Therefore, it could be easier to identify infected people and place them in isolation, stopping the virus spreading to other people while waiting for the repetition of the PCR test.

Moreover, considering the overload of COVID-19-testing laboratories and the consequent delay in the test results, a CT scan could represent a rapid diagnostic tool to confirm a clinically suspected COVID-19 pneumonia, with practical relevance for the community.

Data of patients with COVID-19 who have cancer have been recorded across the world to identify the impact of cancer and related treatments on COVID19 outcomes and to decide the best oncological treatment strategy.

It has been reported that age, gender, comorbidities, and smoking are potential prognostic factors for mortality and severe illness [9,10,11,12,13], but we did not find any correlation between disease severity and age, sex, smoking, or cardiovascular comorbidities. According to CCC19 report, cancer type, type of anticancer therapy, and recent surgery were not associated with mortality [9], and the UKCCMP reported that mortality from COVID-19 in cancer patients cannot be related to active cytotoxic chemotherapy or other anticancer treatments [10]. These observations were confirmed also by the data analyses at the Gustave Roussy Cancer Centre [13] and at the Memorial Sloan Kettering Cancer Center [12], even if an increased risk for COVID-19 pneumonia severity during treatment with ICI has been stressed.

In our study, patients who were on cancer treatment developed a milder disease than cancer patients who were not on treatment. However, this result should be cautiously considered, as we underline the higher risk of treated hematological patients with suppressed lymphocyte-related immunity.

In this study six patients died; only one of them was on cancer treatment, while the other five were being followed up. Remarkably, six cycles of immunochemotherapy (R-CHOP/R-DHAP) with anti-CD20 monoclonal antibody (Mab) and high-dose glucocorticoids, had been administered to the patient who died on treatment; consequently, lymphocytes count and immunoglobulin levels were much lower than in other patients on treatment. Moreover, anti-CD20 Mab has been known to reactivate certain viral infections [21,22]. The retrospective nature of this work from a single institution and the heterogeneity of our cancer center population are inherent limitations of our study. Multicentric studies are needed to better understand COVID-19 in cancer patients and to help clinicians to decide whether to continue or to stop cancer treatment in the context of COVID-19 risk. Several studies are still ongoing, and preliminary results have already been published [9,10,11,12,13].

Regarding patients who are eligible for allogenic hematopoietic stem cell transplantion (HSCT), in the absence of a marker to predict the clinical course or outcome of COVID-19 [23], finding the best compromise seems reasonable: patients are being treated urgently if a delay would result in a risk of disease progression greater than that of contracting COVID-19. In general, it is recommended that patients who are positive for SARS-CoV-2 should have transplantation delayed until their viral test is negative or for at least 14 days after symptoms removal or their first positive test, according to ASH (American Society of Hematology) recommendations (October 2020).

## 5. Conclusions

The acceptance of a CT-defined diagnosis of COVID-19 in areas with a high incidence of SARS-CoV-2, like Bergamo, highlighted a larger COVID-19 oncological population than that diagnosed using RT-PCR, in particular, asymptomatic and mildly symptomatic patients. Considering the limitations of this retrospective study, we can conclude that we did not find any correlation between disease severity and age, sex, smoking, or cardiovascular comorbidities. Remarkably, we observed that actively treated patients had a milder disease, according to previous studies that suggested a protective role of immunosuppression.

The role of CT in monitoring the progression and complications of COVID-19 pneumonia is well established; however, its role as a potential adjunct for the diagnosis of COVID-19 should be better understood, especially considering that the RT-PCR-based viral nucleic acid test is time-consuming, and laboratories’ testing capacity may be a bottleneck in COVID-19 diagnosis due to the rapidly growing population with suspected COVID-19. Moreover, highly suspicious CT imaging features can identify infected patient with initial false-negative or weakly positive RT-PCR test results.

Cases of COVID-19-positive patients detected by RT-PCR with initially normal chest CT findings and cases of patients with initial false-negative RT-PCR test results but characteristic pneumonia features on CT have been reported. The exact reason of these discrepancies is not clear and is still under investigation. Even if these cases are a small proportion of the infected population, it is essential not to underestimate their impact on occult infection transmission.

## Figures and Tables

**Table 1:** Characteristics of cancer patients with coronavirus disease 2019 (COVID-19). GGO, COPD, HBV, HCC, NHL, DLBCL, MCL, R-CHOP/R-DHAP, CLL; HD, ICU.

Patient	Sex	Age	CT Features	Cancer Type	Chemotherapy or Follow Up	Radiotherapy	Setting	Comorbidities	Smoking	SIAARTI Stage	Hospital Admission	Nasofaringeal Swabbing	Outcome
B.G.	F	74.8	GGO (24 March 2020)	Pancreas	Gemcitabine-Abraxane		T2 N + M0	Diabetes mellitus; Hypertension	former	Asymptomatic	No	Negative (30 March 2020)	Alive
B.V.	M	56.8	GGO (28 April 2020)	Colon	FOLFIRI–Bevacizumab		Advanced	No	No	II (fever)	No	Negative (27 April 2020)	Alive
B.M.	F	53.5	GGO (resolving) 28 April 2020	Breast	Follow-up		T2N3M0	No	Former	Asymtomatic	No	No	Alive
B.G.	M	73.0	GGO (12 March 2020)	Abdominal mesothelioma	Carboplatin–pemetrexed		Advanced, progression of disease	Dyslipidemia, previous colon cancer	No	III (fatigue, anorexia, abdominal pain)	No	Negative (12 March 2020)	Alive
C.M.	F	61.01	GGO (7 February 2020)	Colon + breast	FOLFOX; Everolimus + exemestane ongoing		T3 N1b M0 (colon); IV stadio (breast)	No	Former	Asymptomatic	No	No	Alive
F.G.	F	72.1	GGO (6 February 2020)	Breast	Follow-up		T1bN0	Diabetes mellitus; Hypertension; ischemic cardiomiopathy	Former	Asymtpomatic	No	No (household members affected by COVID-19)	Alive
F.T.	F	79.52	GGO (12 March 2020)	Colon and Breast		Yes	Advanced (colon); T2N0 (breast)	Pulmonary Embolism; Hypertension; Congestive heart failure; Chronic kidney disease	No	Asymptomatic	No	No	Alive
G.M.	F	74.5	GGO and left pulmonary consolidation (27 April 2020)	Breast	Carboplatin		Advanced	Pulmonary embolism; dyslipidemia	No	II (cought)	No	No (symptoms referred after remission)	Alive
M.M.	F	58.02	GGO (1 February 2020)	Oesophageal	FLOT		Locally advanced (neoadjuvant)		Si	Asymptomatic	No	No	Alive
M.T.	F	70.3	GGO (14 March 2020)	Breast	Paclitaxel–Bevacizumab	20 Gy (bone metastasis)	T2 N0 M1	Pulmonary embolism	former	III (fever dyspnea)	Yes	Positive (14 March 2020)	Alive
S.G.	M	82.15	GGO and pulmonary consolidation (20 April 2020)	Urothelial and colon	FOLFOX		T3N2b (colon) adjuvant	Hypertension, COPD	Former	II (fever)	No	Positive (20 April 2020)	Alive
S.G.	M	68.6	GGO (6 April 2020)	Bone metastasis of gastric cancer		20 Gy (bone metastasis)	Advanced	Pulmonary embolism		Asymptomatic	yes for pulmonary embolism	Negative (16 April 2020) 10 days after CT scan)	Alive
S.O.	F	41.95	GGO and pulmonary consolidation (17 March 2020)	Breast	Adriamycine-cyclophosphamide–taxol		T4 N2bM0 (neoadjuvant)	No	No	Asymptomatic	No	Positive (24 March 2020)	Alive
T.E.	M	56.8	GGO (1 April 2020)	Pancreas	FOLFIRINOX		T4N1	Hypertension	No	II (fever, cought)	No	Positive (2 April 2020)	Alive
T.A.	F	70.8	GGO (28 February 2020)	Breast	Adriamycine–cyclophosphamide		T1c N0 M0 (adjuvant)	HBV-related HCC; hypothyroidism, previous gastric cancer; coeliac disease; COPD	former	Asymptomatic	No	No	Alive
V.G.	M	76.3	GGO and pulmonary consolidation (17 March 2020)	NHL DLBCL	Follow-up		I A Ann Arbor	Hypertension, rheumatoid arthritis	No	Asymptomatic	No	No	Alive
V.M.	M	51.6	GGO (7 April 2020)	Head and neck	Cisplatin	Yes	T3 N0/1 M0	HCV	Yes	Asymptomatic	No	Negative (17 April 2020)	Alive
Z.L.	F	56.7	GGO (26 March 2020)	NHL MCL	R-CHOP/R-DHAP		IV Ann Arbor	Previous choroidal melanoma	No	III → VI (fever and cough at the onset)	Yes ICU	Positive (26 March 2020)	Dead
S.G.	M	74.3	GGO (2 April 2020)	Prostatic	Follow-up		Not followed in our hospital	Hypovitaminosis D	No	III (fever, cought)	Yes	Positive (2 April 2020)	Alive
R.R.	F	65.47	patchy shadowing pulmonary consolidation (8 April 2020)	Head and neck	Carboplatin	Yes	T1N3b adjuvant. Not followed in our hospital	Hypertension, COPD, peripheral arterial disease, pulmonary embolism	Former	III (dyspnea, cough) concomitant pulmonary embolism	Yes	**Negative** (8 April 2020)	Alive
M.L.	F	78.15	GGO (19 March 2020)	Breast	Follow-up		Not followed in our hospital	Hypertension, radiation induced pulmonary fibrosis	No	III → VI (fever, dyspnea, cough at the onset)	Yes ICU	Positive (19 March 2020)	Dead
B.A.	F	72.27	GGO (16 March 2020)	Breast	Follow-up		T1cN0	Hypertension, Diabetes mellitus, Dyslipidemia	No	III (fever, dyspnea)	Yes	Positive (16 March 2020)	Alive
C.M.	F	77.4	GGO, pulmonary consolidation (30 March 2020)	Breast	Follow-up		Advanced	Hypertension	No	II → VI (nausea, fever, anorexia)	Yes ICU	Positive (30 March 2020)	Dead
R.M.	M	78.3	GGO (16 March 2020)	Urothelial	Follow-up		Not followed in our hospital	Hypertension, COPD, congestive heart failure, atrial fibrilation, ictus	No	III → VI (nausea, fever, anorexia)	Yes	Positive (16 March 2020)	Dead
N.G.	F	86.7	Bilateral interstitial abnormalities; pulmonary consolidation (6 April 2020)	CLL	Watch-and-wait follow up (never treated)			Hypertension, diabetes mellitus	No	III → VI (fever, cough, dyspnea, asthenia)	Yes	Positive (6 April 2020)	Dead
M.L.	F	62.29	GGO (4 April 2020)	HD	ABVD 6 cycles		IV b Ann Arbor. Not followed in our hospital	Previous gynecological cancer	No	III (fever)	Yes	Positive (4 April 2020)	Alive
S.M.	F	75.13	GGO (30 March 2020)	Breast	Follow-up		Not followed in our hospital	Hypertension, atrial fibrillation, diabetes mellitus	No	III → VI (fever, asthenia, cough)	Yes	Positive (30 March 2020)	Dead
G.C.	F	92.54	GGO (30 April 2020)	Gastric	Follow-up		T3N0	Previous breast cancer, hypertension, chronic gastric reflux	No	III (dyspnea) concomitant atrial fibrillation and congestive heart failure	Yes	**Negative** (30 April 2020)	Alive
C.E.	M	56.5	GGO and pulmonary consolidations (9 April 2020)	CLL	Follow-up		Not followed in our hospital	No	No	III (fever, cought, dyspnea)	Yes	Positive (9 April 2020)	Alive
M.L.	M	85.79	GGO (31 March 2020)	Lung	Follow-up		Advanced, Progression disease. Not followed in our hospital	Dyslipidemia, hypertension, COPD, chronic renal disease	Former	III (dyspnea, fever)	Yes	Positive (31 March 2020)	Alive
F.N.	F	54	**Negative** (14 March 2020)	Breast	Adriamycine–cyclophosphamide		Locally advanced (neoadjuvant)	No	No	II (fever)	No	Positive (3 March 2020)	Alive
S.A.	F	40	**Negative** (2 April 2020) CT scan after symptoms’ remission	Breast	Trastuzumab		T2N1M1	Hypothyroidism, multiple sclerosis	No	II (fever, cought)	No	Positive (20th March 2020)	Alive

GGO: ground-glass opacities; COPD: Chronic Obstructive Pulmonary Disease; HBV: Hepatitis B virus; HCC: Hepatocellular carcinoma; NHL: Non Hodgkin Lymphoma; DLBCL: Diffuse Large B Cell Lymphoma; MCL: Mantle Cell Lymphoma; R-CHOP/R-DHAP: Rituximab–Cyclophosphamide–Hydroxydaunorubicin–Oncovin–Prednisone/Rituximab–Dexamethasone–Ara-C–Cisplatin; CLL: Chronic Lymphocytic Leukemia; HD: Hodgkin Disease; ICU: intensive care unit.

## Data Availability

The datasets used and/or analyzed during the current study are available from the corresponding author on reasonable request.

## List of Abbreviations

CT	computed tomography
RT-PCR	Real-time polymerase chain reaction
COVID-19	coronavirus disease 19
GGO	ground-glass opacities
CV	cardiovascular
OR	odds ratio
R-CHOP/R-DHAP	Rituximab–Cyclophosphamide–Hydroxydaunorubicin–Oncovin–Prednisone/Rituximab–Dexamethasone–Ara-C–Cisplatin
Mab	monoclonal antibody
